# Quantitative flow ratio for assessment of donor vessel in patients with chronic total occlusions

**DOI:** 10.1186/s12872-026-05728-6

**Published:** 2026-03-12

**Authors:** Xinjian Li, Juntao Duan, Lin Mi, Liyuan Tao, Xinye Xu, Guisong Wang

**Affiliations:** 1 Department of Cardiology and Institute of Vascular Medicine, Peking University Third Hospital, Beijing, China; 2 State Key Laboratory of Vascular Homeostasis and Remodeling, Peking University, Beijing, China; 3 NHC Key Laboratory of Cardiovascular Molecular Biology and Regulatory Peptides, Peking University, Beijing, China; 4 Beijing Key Laboratory of Cardiovascular Receptors Research, Beijing, China; 5 Clinical Epidemiology Research Center, Peking University Third Hospital, Beijing, China

**Keywords:** Chronic total occlusions, Quantitative flow ratio, Donor vessel

## Abstract

**Background:**

Chronic total occlusion (CTO) is a challenging subset of coronary artery disease characterized by complete vessel obstruction and development of collateral circulation. In CTO patients, donor vessels supplying collateral flow may be at risk of functional ischemia. Functional assessment of these donor vessels can provide valuable insights into the ischemic status. Quantitative flow ratio (QFR) is an angiography-based method for the functional assessment of coronary arteries and Murray-law based QFR (µQFR) requires only a single coronary projection, enhancing its applicability. This study primarily aims to investigate the agreement between µQFR and 3D-QFR for functional assessment of donor vessels in CTO patients.

**Methods:**

This single-center retrospective study enrolled patients with a single-vessel CTO lesion between January 2015 and June 2023. Both µQFR and 3D-QFR were used to assess the function of the predominant collateral donor artery following standard protocol.

**Results:**

A total of 336 CTO patients were included. There was a good correlation between µQFR and 3D-QFR (*r* = 0.947, *p* < 0.001). The diagnostic accuracy was 96.1% (95% CI, 93.5%–97.9%). ROC curve analysis revealed an AUC of 0.984 (95% CI, 0.971–0.997) for µQFR in estimating 3D-QFR.

**Conclusions:**

µQFR has good correlation and agreement with standard 3D-QFR for the functional assessment of donor vessels in CTO patients.

## Introduction

Coronary chronic total occlusions (CTO) are found in approximately one-fourth of patients with obstructive coronary artery disease [1]. In these patients, collateral circulations often develop from donor vessels, supplying blood to the ischemic myocardium [2]. The vessel supplying the largest collateral contribution is referred to as predominant collateral donor artery (PCDA) [3]. The maximal blood flow in a coronary artery is proportional to the mass of viable myocardium it supplies [4]. Once collateral circulation forms, the area of myocardium supplied by the donor vessel increases, which may lead to ischemia, particularly when the donor vessel itself has stenotic lesions [5, 6]. Currently, guidelines recommend functional assessment to guide revascularization in stable coronary artery disease [7, 8]. Therefore, functional assessment of donor vessels is an important topic of clinical interest.

Quantitative flow ratio (QFR) is an angiography-derived method for the functional assessment of coronary arteries, which does not require pressure wire or hyperemia induction and has demonstrated high diagnostic accuracy in identifying hemodynamically significant coronary stenosis [9–11]. The FAVOR (Functional Assessment by Virtual Online Reconstruction) III China trial demonstrated that QFR-guided revascularization significantly reduced major cardiovascular events compared to angiography-guided revascularization [12]. The 2024 ESC Guidelines for the management of chronic coronary syndromes recommend the use of QFR for evaluating the function of intermediate lesions, and commend its application in the context of multivessel coronary artery disease [7].

The calculation of QFR typically requires two angiographic projections for 3-dimensional (3D) vessel reconstruction, which may limit its feasibility, particularly in retrospective studies [13]. A few studies have explored the use of 3D-QFR for functional assessment of collateral donor vessels [14, 15]. However, patients with CTO often present with complex coronary anatomy, and obtaining two angiographic projections separated by at least 25° is sometimes challenging in clinical practice. Murray law-based QFR (µQFR) is a novel QFR method that requires only a single angiographic projection, potentially improving feasibility in CTO patients [16]. Previous studies have investigated the agreement between µQFR and 3D-QFR in general coronary lesions [17]. However, data on the agreement between µQFR and 3D-QFR in collateral donor vessels remain limited. Therefore, the present study aimed to explore the agreement between µQFR and 3D-QFR for functional assessment of donor vessels in patients with CTO.

## Methods

### Study population

Patients with a single-vessel CTO diagnosed by coronary angiography were retrospectively enrolled at Peking University Third Hospital between January 2015 and June 2023. CTO was defined as a complete occlusion with TIMI (Thrombolysis In Myocardial Infarction) flow grade 0 for at least 3 months in a major coronary artery with a diameter of at least 2.5 mm [18]. Patients included in this study had intermediate stenosis in PCDA by visual evaluation (30–70%) with visible retrograde collateral flow (Rentrop grade ≥ 1) [19]. Exclusion criteria included ipsilateral collateral channels, bypass grafts, left main coronary artery disease, myocardial infarction, arrhythmia, severe overlap or tortuosity, lack of a second angiographic projection separated by at least 25°, and poor image quality unsuitable for QFR measurement. The study was conducted in accordance with the Declaration of Helsinki for investigations involving human subjects and was approved by the Ethics Committee of Peking University Third Hospital, with a waiver of informed consent due to the retrospective nature of the study and the use of anonymized data.

### µQFR and 3D-QFR analysis

**Fig. 1:**
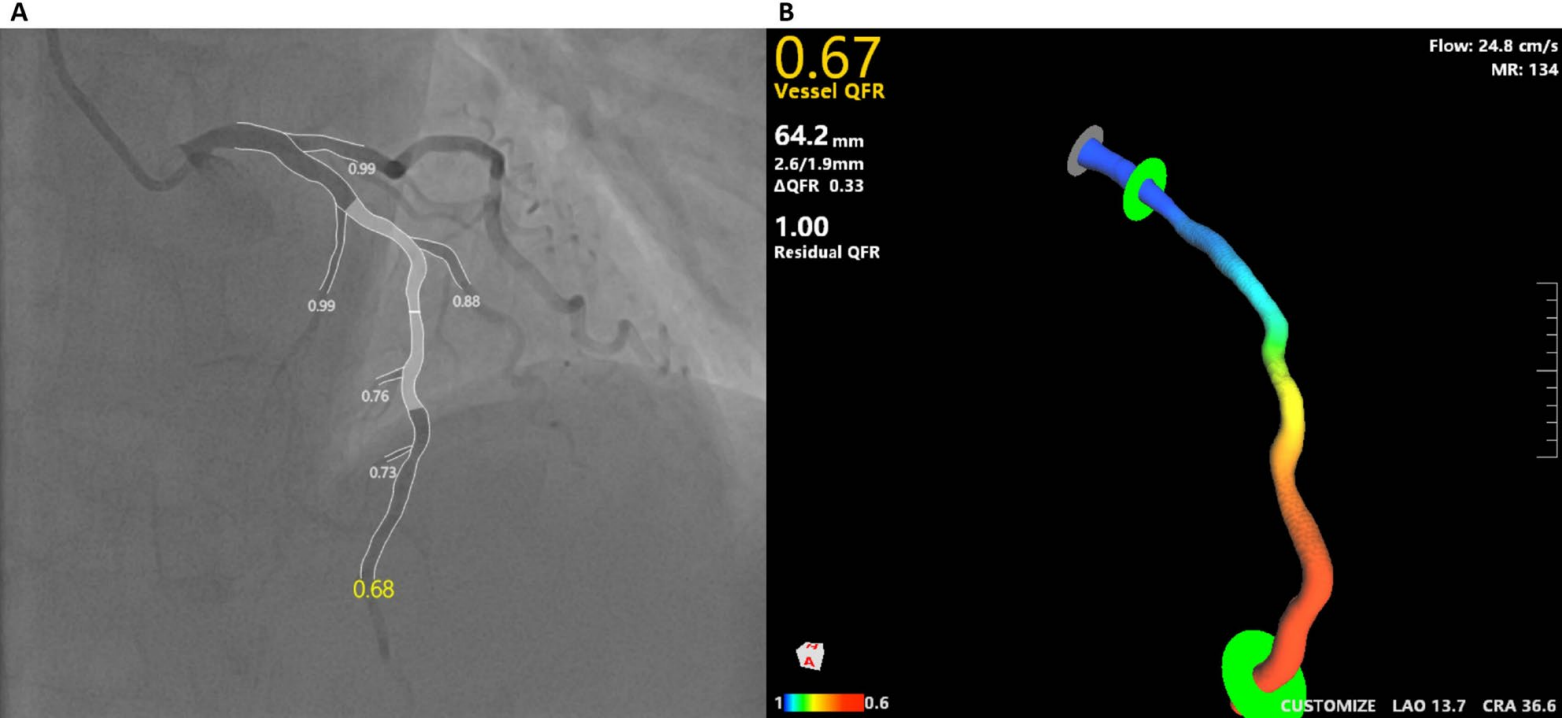
A representative example of analysis by µQFR and 3D-QFR This figure illustrates a representative case analyzed using two quantitative flow ratio modalities: Murray law-based quantitative flow ratio (µQFR) (**A**) and three-dimensional quantitative flow ratio (3D-QFR) (**B**)

The µQFR and 3D-QFR were retrospectively analyzed by certified operators using validated software (AngioPlus Core, Pulse Medical Imaging Technology, Shanghai, China) according to standard operating procedures [16, 20]. The operators performing 3D-QFR analysis were blinded to the corresponding µQFR results for the same vessel. For µQFR, a single angiographic projection was selected, with optimal lesion exposure, minimal vessel overlap and foreshortening. The lumen contour of the target vessel and side branches was automatically delineated, and the contrast flow velocity was automatically calculated. A frame with good contrast filling and full exposure of the lumen contour was selected as the analysis frame. The operator could adjust the contour and frame if needed. The pressure drop was calculated based on fluid dynamic equations, and the µQFR value was divided. For 3D-QFR, two optimal angiographic projections separated by at least 25° were selected for analysis. A 3D reconstruction of the interrogated vessel was performed, and the lumen and reference vessel sizes were calculated from the reconstruction data. The 3D-QFR was calculated based on fluid dynamic equations. A representative example of µQFR and 3D-QFR analysis is shown in Fig. 1. Both 3D-QFR and µQFR values ≤ 0.80 were defined as indicating functional ischemia.

### Statistical analysis

Continuous variables are presented as mean ± standard deviation (SD) or median (Q1–Q3) depending on their distribution, and comparisons were made using Student’s t-test or the Mann-Whitney test as appropriate. Normality was assessed using the Shapiro–Wilk test. Categorical variables are reported as frequencies and percentages, with comparisons made using the Pearson chi-square test or Fisher’s exact test as applicable. The correlation between µQFR and 3D-QFR was assessed using Pearson correlation coefficient (r). Bland-Altman analysis was performed to evaluate the agreement between continuous variables. Intraclass correlation coefficient (ICC) analysis was used to assess agreement between µQFR and 3D-QFR. Receiver operating characteristic (ROC) curve analysis and the area under the curve (AUC) were used to evaluate the accuracy of µQFR in predicting 3D-QFR of ≤ 0.80. Sensitivity, specificity, positive predictive value, negative predictive value, positive likelihood ratio, negative likelihood ratio, and diagnostic accuracy were also reported. All analyses were conducted using R, version 4.4.1.

## Results

### Baseline characteristics

**Fig. 2:**
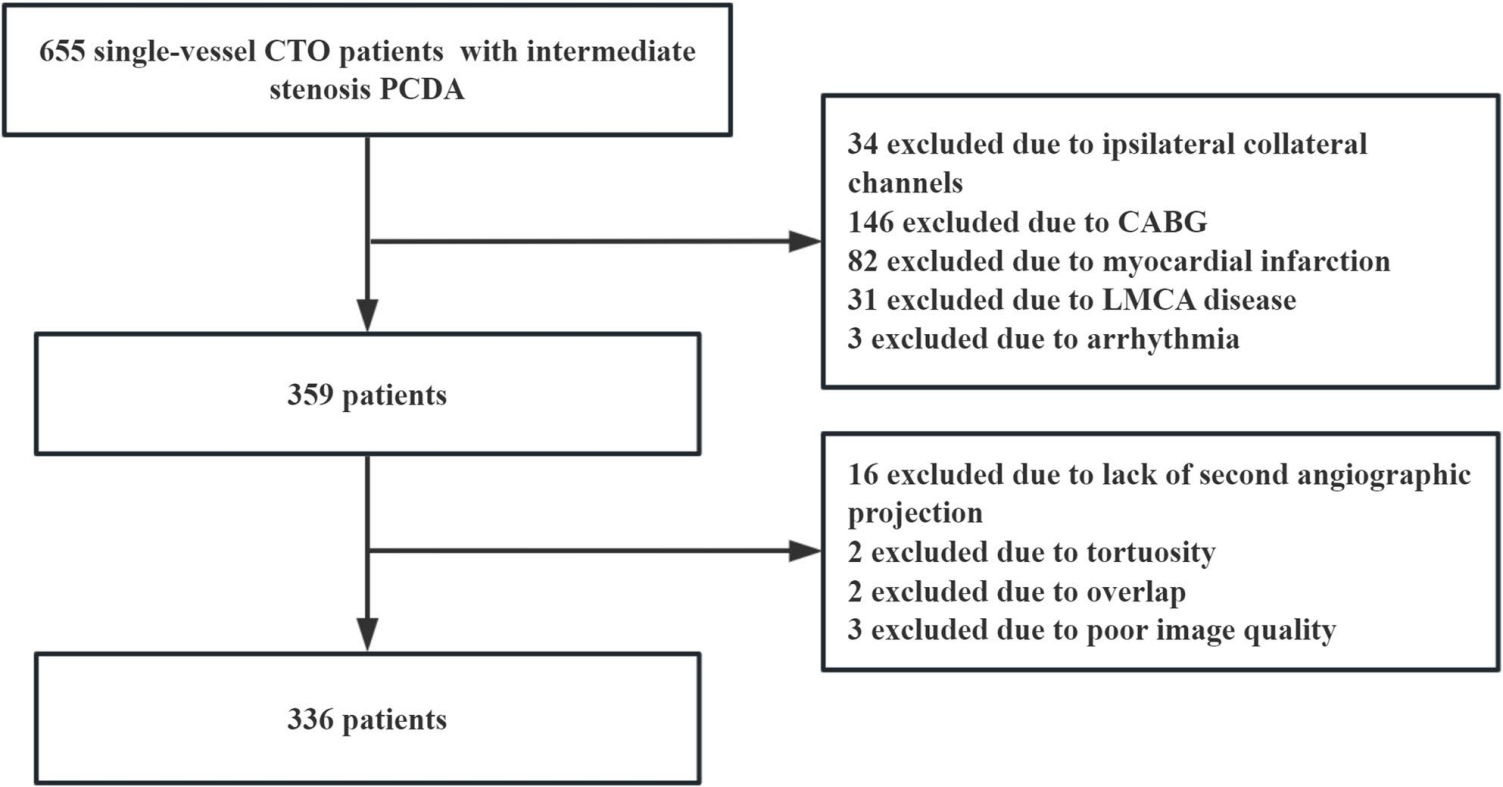
Flowchart of participants selection Abbreviations: CTO, chronic total occlusion; PCDA, predominant collateral donor artery; CABG, coronary artery bypass grafting; LMCA disease, left main coronary artery disease

A total of 336 patients were included in this study. Twenty-three donor vessels were not suitable for 3D-QFR analysis, with 16 cases due to the lack of a second angiographic projection (Fig. 2). Baseline characteristics are summarized in Table 1. The mean age was 60.8 ± 10.4 years, and 81.2% of the participants were male. Among the CTO lesions, 145 (43.2%) were located in the left anterior descending artery (LAD), 43 (12.8%) in the left circumflex artery (LCX), and 148 (44.0%) in the right coronary artery (RCA). In terms of PCDA, the LAD accounted for 121 (36.0%), the LCX for 56 (16.7%), and the RCA for 159 (47.3%). Among the collateral channels, 137 (40.8%) were epicardial, and 199 (59.2%) were septal. Additionally, 112 (33.3%) of the collateral channels had a Rentrop score > 2.

**Table 1:** Baseline Characteristics of Patients and Lesions

Variables	*N* = 336
Age [years], mean ± SD	60.8 ± 10.4
Male, n (%)	273 (81.2%)
Cardiovascular risk factors, n (%)	
Diabetes mellitus	121 (36.0%)
Hypertension	236 (70.2%)
Current smoker	138 (41.1%)
Hyperlipidemia	154 (45.8%)
Previous PCI, n (%)	101 (30.1%)
Previous stroke, n (%)	43 (12.8%)
Family history, n (%)	70 (20.8%)
Glycated hemoglobin [%], mean ± SD	6.6 ± 1.3
LDL cholesterol [mmol/L], median (IQR)	2.1 (1.7, 2.7)
LVEF [%], mean ± SD	65.4 ± 9.0
Location of CTO lesions, n (%)	
Left anterior descending artery	145 (43.2%)
Circumflex artery	43 (12.8%)
Right coronary artery	148 (44.0%)
Location of PCDA lesions, n (%)	
Left anterior descending artery	121 (36.0%)
Circumflex artery	56 (16.7%)
Right coronary artery	159 (47.3%)
Collateral channel	
Epicardial	137 (40.8%)
Septal	199 (59.2%)
Rentrop score	
≤ 2	224 (66.7%)
> 2	112 (33.3%)
3D-QFR, mean ± SD	0.9 ± 0.1
µQFR, mean ± SD	0.9 ± 0.1

*Abbreviations*: *SD* Standard deviation, *IQR* Interquartile range, *PCI* Percutaneous coronary intervention, *LDL* low-density lipoprotein, *LVEF* Left ventricular ejection fractions, *CTO* Chronic total occlusion, *PCDA* Predominant collateral donor artery, *3D-QFR* 3-dimensional quantitative flow ratio, *µQFR* Murray law-based quantitative flow ratio

### Correlation and agreement between µQFR and 3D-QFR

**Fig. 3:**
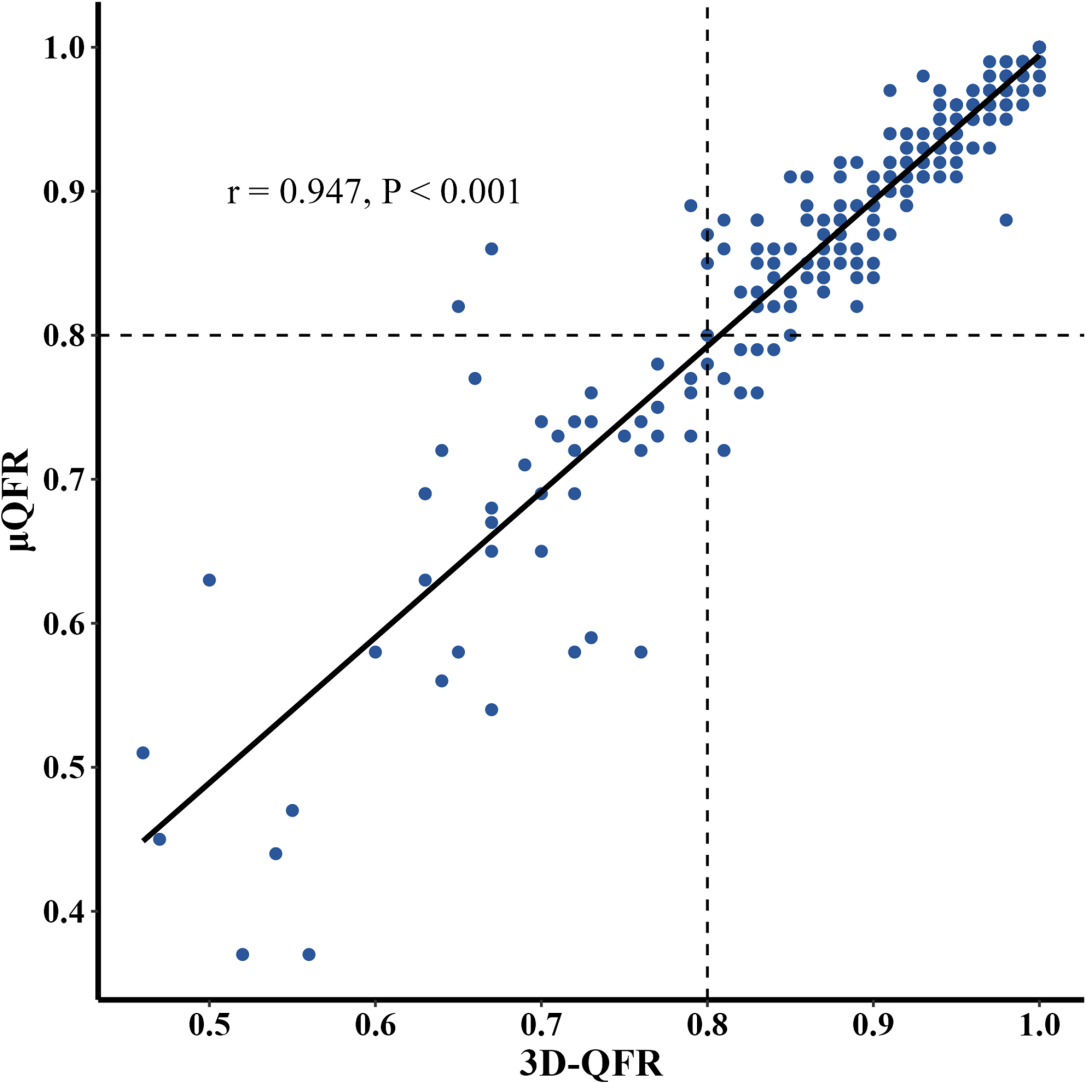
Correlation of µQFR and 3D-QFR A good correlation was observed between μQFR and 3D-QFR in donor vessels of CTO patients. Abbreviations: 3D-QFR, 3-dimensional quantitative flow ratio; μQFR, Murray law-based quantitative flow ratio; CTO, chronic total occlusion

**Fig. 4:**
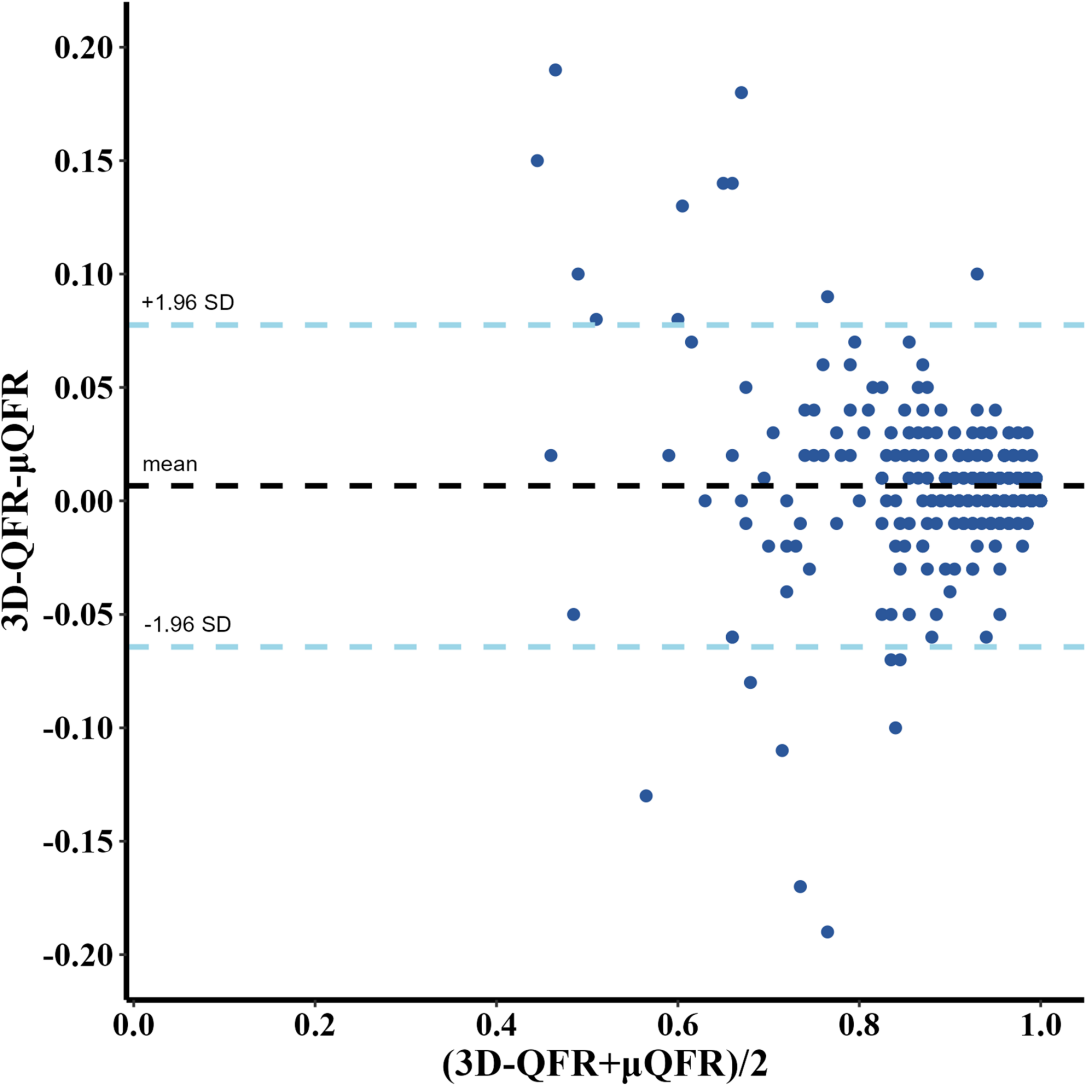
Bland–Altman plot of µQFR and 3D-QFR A good agreement was observed between µQFR and 3D-QFR in donor vessels of CTO patients. Abbreviations: µQFR, Murray law-based quantitative flow ratio; 3D-QFR, 3-dimensional quantitative flow ratio; CTO, chronic total occlusion

The mean values for both 3D-QFR and µQFR were 0.9 ± 0.1. As shown in Fig. 3, µQFR and 3D-QFR demonstrated significant positive correlation (*r* = 0.947, *p* < 0.001). Bland-Altman analysis revealed a mean difference of 0.007 (-0.064 to 0.078) between the two methods (Fig. 4). When analyzed as dichotomous variables, intraclass correlation coefficient analysis showed good agreement between µQFR and 3D-QFR (ICC = 0.943 [95% CI, 0.929–0.954]).

### Diagnostic performance

**Fig. 5:**
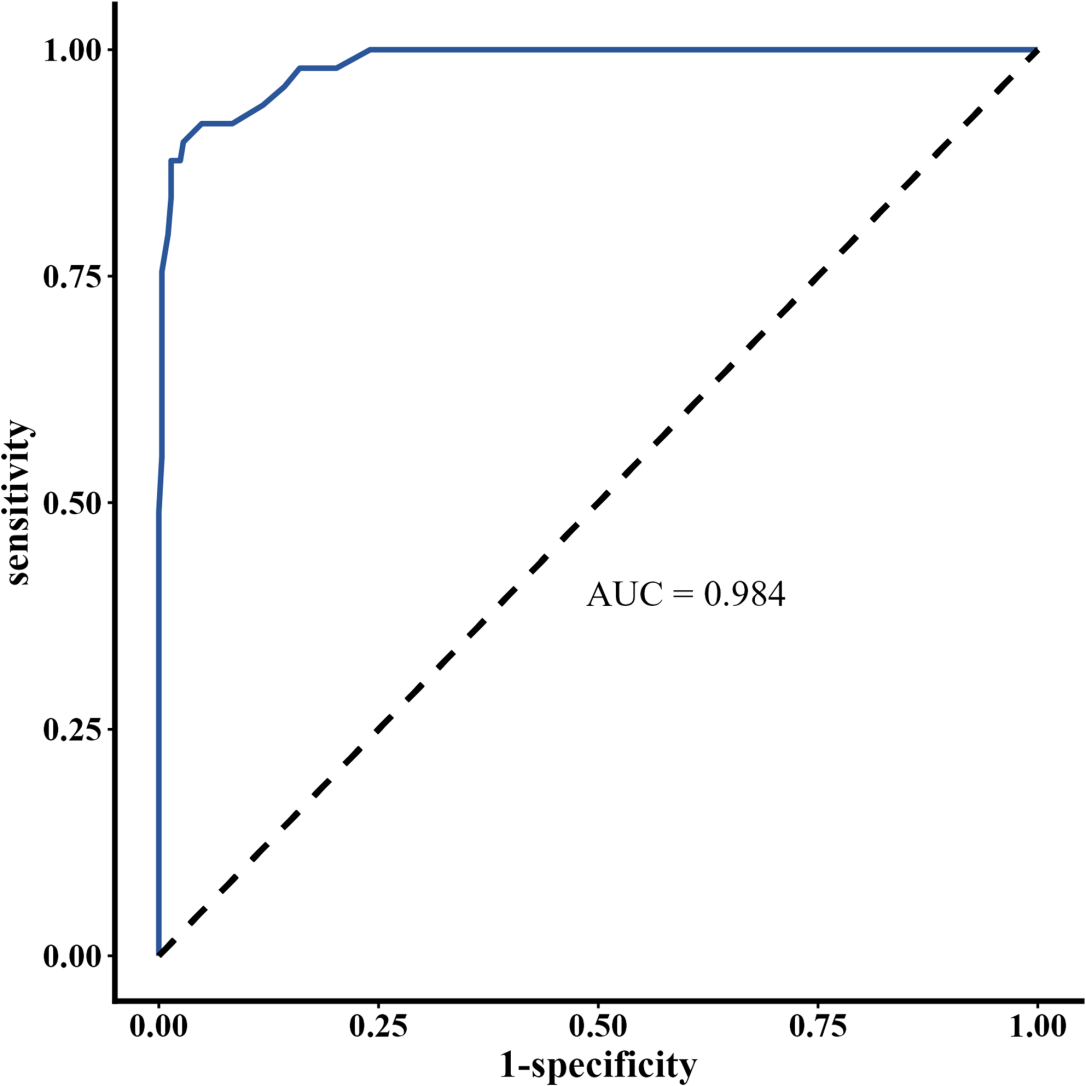
Receiver operating characteristic curve of µQFR and 3D-QFR µQFR presented a good diagnostic efficiency in detecting functional ischemia according to 3D-QFR. Abbreviations: AUC, area under the curve; µQFR; Murray law-based quantitative flow ratio; 3D-QFR, 3-dimensional quantitative flow ratio

The diagnostic accuracy was 96.1% (95% CI, 93.5%-97.9%). Eight vessels (2.4%) had µQFR ≤ 0.80 while 3D-QFR > 0.80 (false positives), and five vessels (1.5%) had µQFR > 0.80 while 3D-QFR ≤ 0.80 (false negatives). The sensitivity, specificity, positive predictive value, and negative predictive value were 89.8%, 97.2%, 84.6%, and 98.2%, respectively (Table 2). ROC curve analysis showed that the AUC of µQFR for estimating 3D-QFR was 0.984 (95% CI, 0.971–0.997), as illustrated in Fig. 5. The smoothness of the ROC curve is due to the high diagnostic accuracy, leading to minimal variation in the results.

**Table 2:** Diagnostic performance of µQFR for predicting 3D-QFR

	Value	95% CI
Sensitivity	89.8%	77.8%-96.6%
Specificity	97.2%	94.6%-98.8%
Positive predictive value	84.6%	71.9%-93.1%
Negative predictive value	98.2%	95.9%-99.4%
Diagnostic accuracy	96.1%	93.5%-97.9%
Positive likelihood ratio	32.2	16.2, 64.2
Negative likelihood ratio	0.11	0.05, 0.24

*Abbreviations*: *3D-QFR* 3-dimensional quantitative flow ratio; *µQFR* Murray law-based quantitative flow ratio

## Discussion

This study primarily investigates the correlation and agreement between µQFR and 3D-QFR in the predominant collateral donor arteries of CTO patients. The results show good agreement between µQFR and 3D-QFR, suggesting that µQFR can reliably assess donor vessel function, particularly when obtaining two suitable angiographic projections for 3D-QFR analysis is challenging.

Since the FAME study, functional assessments such as fractional flow reserve (FFR) have become increasingly important in guiding coronary interventions. QFR, a wireless functional assessment tool, has demonstrated good agreement with wire-based functional assessments [16]. It offers the advantage of not requiring a pressure wire or pharmacologic hyperemia induction, making it faster and easier to perform. µQFR, a new method for assessing QFR, requires only a single angiographic projection and less assessment time [16]. Previous studies have shown that µQFR and 3D-QFR have good agreement in patients with chronic coronary syndromes [17]. However, CTO patients are often excluded from these studies. Our study investigates the agreement between µQFR and 3D-QFR in the predominant donor vessels of CTO patients, thereby expanding the application of these assessments.

Previous functional studies of donor vessels primarily focused on pressure wire-based assessments [21]. FFR requires the use of adenosine and pressure wire. Given the complexity of CTO lesions, the treatment process can be time-consuming and costly, and measuring FFR in donor vessels prior to CTO revascularization would further increase procedure time and costs. Moreover, after successful recanalization of the CTO, FFR values may slightly increase [22]. Therefore, performing wire-based functional assessment of moderately stenosed donor vessels prior to CTO recanalization may be limited and could increase the potential risk of complications.

In contrast, QFR does not require the pressure wire and involves a shorter measurement time, allowing for immediate functional assessment following coronary angiography. Moreover, Dan et al. [15] reported using 3D-QFR for the functional evaluation of the donor vessel to minimize potential injury to the donor vessel. Although QFR, like FFR, may slightly increase after successful CTO revascularization [14], it allows for an initial functional assessment with minimal time and cost. If significant ischemia is detected, ischemia may still persist even if there is a slight increase in QFR after successful CTO recanalization [23]. Functional assessment of the collateral vessels using µQFR can be performed prior to CTO revascularization. If CTO revascularization is successfully achieved, the donor vessels with functional ischemia should be re-evaluated at a later time to assess whether ischemia persists. If CTO revascularization is unsuccessful, interventional treatment may be considered for donor vessels with functional ischemia.

µQFR, which requires only a single angiographic projection, demonstrates good agreement with 3D-QFR and can serve as an important tool for the functional assessment of donor vessels in CTO patients. Approximately 12% of cases and 3% in prospective studies are unable to undergo 3D-QFR analysis in retrospective studies. The inability to obtain two high-quality angiographic projections with an angle ≥ 25° is a common reason [13, 17]. This makes µQFR especially suitable when obtaining a second appropriate projection is challenging due to vessel foreshortening, tortuosity, or overlap. Consequently, common scenarios for the use of µQFR include: when FFR cannot be obtained due to comorbidities such as asthma, when FFR is inconvenient due to its potential to extend procedure time or increase costs, or when obtaining a second suitable angiographic projection is difficult.

### Limitation

This study has several limitations. First, as a retrospective study, the angiographies were not specifically obtained for QFR analysis, so the feasibility of QFR assessment may be further improved in prospective clinical applications. Second, although standardized analysis procedures were applied, angiography-derived functional assessment may still be influenced by operator-dependent variability and boundary conditions [24]. Third, although the agreement between µQFR and FFR has been well established in previous studies, a direct comparison between µQFR and FFR was not performed in this study. Finally, as this study focused on donor vessels in patients with CTO, the generalizability of our findings to other clinical settings, such as acute coronary syndrome, requires further investigation.

## Conclusions

µQFR has good correlation and agreement with standard 3D-QFR functional assessment of donor vessels in CTO patients. µQFR can be considered a reliable alternative when obtaining two suitable angiographic projections is challenging.

## Data Availability

The data underlying this article will be shared on reasonable request to the corresponding author. The data are not publicly available due to privacy or ethical restrictions.

## Abbreviations

CTO: Chronic total occlusion
QFR: Quantitative flow ratio
μQFR: Murray-law based QFR
3D-QFR: 3-dimensional QFR
PCDA: Predominant collateral donor artery
TIMI flow grade: Thrombolysis In Myocardial Infarction flow grade
ROC: Receiver operating characteristic
AUC: Area under the curve
LAD: Left anterior descending artery
LCX: Left circumflex artery
RCA: Right coronary artery
FFR: Fractional flow reserve
CCS: Chronic coronary syndromes
